# Comparison of 128-Slice Low-Dose Prospective ECG-Gated CT Scanning and Trans-Thoracic Echocardiography for the Diagnosis of Complex Congenital Heart Disease

**DOI:** 10.1371/journal.pone.0165617

**Published:** 2016-10-27

**Authors:** Guilin Bu, Ying Miao, Jingwen Bin, Sheng Deng, Taowen Liu, Hongchun Jiang, Weiping Chen

**Affiliations:** 1 Radiology Department, Nanxi Hill Hospital of Guangxi Zhuang Autonomous Region, Guilin, China; 2 Cardio-Thoracic Surgery, Nanxi Hill Hospital of Guangxi Zhuang Autonomous Region, Guilin, China; 3 Science and Education Department, Nanxi Hill Hospital of Guangxi Zhuang Autonomous Region, Guilin, China; Providence VA Medical Center, UNITED STATES

## Abstract

**Objective:**

To compare prospective ECG-gated multi-slice computed tomography (MSCT) and trans-thoracic echocardiography (TTE) in the diagnosis of complex congenital heart disease (CHD).

**Methods:**

This was a prospective study of consecutive patients with complex CHD (age <7 years) treated at a tertiary hospital between May 2013 and May 2015. All patients were imaged with TTE and prospective ECG-gated 128-slice spiral CT in the week before surgery. Effective radiation dose (ED) was calculated from volume CT dose index (CTDIvol) and dose length product (DLP). Image quality (5-point scale) was assessed independently by two radiologists. Using surgical findings as the reference, the diagnostic capabilities of MSCT and TTE were compared.

**Results:**

Thirty-five patients (19 males) aged 1.59±1.58 years (range, 3 days to 74 months) were included. CTDIvol, DLP and ED were 0.90±0.24 mGy, 12.9±4.7 mGy∙cm and 0.64±0.21 mSv (range, 0.358–1.196 mSv), respectively. Image quality score was 4.3±0.5, and all images met the diagnostic requirements. The sensitivity, specificity, positive predictive value, and negative predictive value for diagnosing CHD were 97.2%, 99.8%, 99.0%, and 99.5%, respectively, for MSCT, and 90.6%, 99.8%, 99.0%, and 98.4%, respectively, for TTE. MSCT not only had a higher sensitivity than TTE overall (97.2% vs. 90.6%; P<0.05), but was much more sensitive for the diagnosis of extracardiac vascular abnormalities (92.0% vs. 68.0%; P<0.05).

**Conclusion:**

128-slice low-dose prospective ECG-gated CT scanning has important clinical value in the diagnosis of complex CHD in children, complementing and extending the findings of TTE.

## Introduction

Congenital heart disease (CHD) is the most commonly occurring congenital anomaly, affecting 4-10/1000 live births [1]. Over the past few decades, advances in diagnostic techniques and surgical management have greatly improved patient survival, with around 90% of patients with CHD reaching adulthood [2]. Complex cases of CHD present with multiple malformations, and require accurate preoperative diagnosis to enable the appropriate planning of surgery. A variety of clinical investigations are currently available for the diagnosis of CHD, including echocardiography, catheter angiography, magnetic resonance imaging (MRI) and computed tomography (CT) [2–4]. Each of these techniques has advantages and disadvantages in the diagnosis of CHD.

Although catheter angiography was used for many years as the investigation of choice for diagnosing CHD, the risk of complications associated with its invasive nature and use of contrast medium led to it being superseded by newer, non-invasive modalities. Trans-thoracic echocardiography (TTE) is now well established as the first-line diagnostic investigation for CHD, due to its non-invasive nature, convenience, and ability to delineate cardiac morphology as well as measure flow velocities using the Doppler technique [5–7]. However, TTE is operator dependent, and has several limitations in the visualization of complex anomalies of extracardiac structures such as the pulmonary arteries, pulmonary veins, aortic arch, and great vessels [3, 4]. Thus, TTE is often inadequate in patients with complex CHD, necessitating the use of complementary approaches. Emerging MRI techniques are able to visualize both intracardiac and extracardiac structures, and have a number of advantages that include functional as well as morphologic evaluation of complex CHD, a multiplanar capability with wide field of view, and no requirement for contrast medium or ionizing radiation [8–10]. However, the disadvantages of MR angiography (MRA) include a lower spatial resolution than other non-invasive techniques, poorer evaluation of the lung tissue and airways, and a long scanning time of 45–60 minutes that increases the risks associated with intubation and anesthesia [3, 4]. Compared with MRA, CT angiography (CTA) is capable of visualizing intracardiac and extracardiac structures (including the airways and lung parenchyma) with higher spatial resolution, has a much shorter scanning time (5–10 minutes), and requires less sedation [3, 4]. Despite these strengths, CTA is limited by its requirements for ionizing radiation and iodinated contrast medium, both of which are associated with risks of complications. The radiation dose associated with conventional CTA (non-ECG-gated or retrospective ECG-gated used in adults for coronary artery imaging) has been estimated to be 12–15 mSv [11, 12], and since children are known to be more radiosensitive than adults [13] it is particularly important that the risks of cancer associated with ionizing radiation be taken into consideration when selecting an imaging modality. As a result, great efforts have been made to develop CT technology in order to substantially reduce the radiation exposure.

Multi-slice spiral computed tomography (MSCT) is a technique that is capable of clearly displaying the morphology of complex CHD [14, 15]. Furthermore, the use of prospective rather than retrospective ECG-gating can reduce the radiation dose associated with this method to 0.2–1.6 mSv [16–23]. The aim of the present study was to determine whether 128-slice low-dose prospective ECG-gated MSCT could be used to accurately diagnose the intracardiac and extracardiac anomalies of children with CHD, while limiting the radiation exposure to levels below 1 mSv. Therefore, we collected and analyzed data for children with complex CHD who had been examined using both 128-slice low-dose prospective ECG-gated MSCT and TTE, in order to compare the clinical utilities of these techniques in the diagnosis of complex CHD.

## Patients and Methods

### Study design

The study was approved by the ethics committee of the Second People’s Hospital of Guangxi Zhuang Autonomous Region, and the parents of all patients provided informed written consent for their inclusion in the study. This was a prospective study of consecutive patients with complex CHD. A total of 35 patients (19 males, 16 females) aged from 3 days to 6 years and 2 months (mean age, 1.59±1.58 years) were included in the study. All patients were treated surgically at the Second People’s Hospital of Guangxi Zhuang Autonomous Region, Guangxi, China, between May 2013 and May 2015. All included patients were examined using both TTE and MSCT within 1 week before surgery.

### Patients

The following inclusion criteria were used for enrolment of patients in the study: 1) age <7 years; 2) more than one pathologic change or multiple cardiovascular abnormalities confirmed during surgery; and 3) imaged with TTE and MSCT in the week before surgery.

### TTE examination

TTE was performed by a sonographer with at least 3 years of experience in ultrasound cardiac diagnosis, using an iE33 xMATRIX Echocardiography System (Philips, Amsterdam, The Netherlands). TTE was carried out using an apical four-chamber view, a left ventricular long-axis view, a right ventricular outflow tract view, and a parasternal short-axis view.

### MSCT scanning technique and parameters

Children who were <4 years old and uncooperative were orally administered 10% chloral hydrate, at a dose of 50 mg/kg, 30 min before MSCT scanning. Children aged 4 years or older were given training in breathing techniques so that scanning could be performed without the use of sedation. Examination started when heart rate was ≤140. Drugs were not used for heart rate control.

MSCT was performed using a SOMATOM Definition AS+ 128-slice spiral CT scanner (Siemens AG, Erlangen, Germany). Axial scanning was conducted using prospective ECG-gating, with the scanning range set from the thoracic inlet to 3 cm below the diaphragm. The delay time was determined by an empirical method (21–23 s). The contrast medium injection velocity was adjusted according to the patient’s weight and the injection time was 13 s for all patients, followed with normal saline at the same velocity for 10 s. After injection of the CM for 21–23 s, CM equally and uniformly filled the left and right ventricles, atria, major arteries, and veins. The delay time was set as 21 s for upper right extremity injection, and as 22–23 s for upper left and lower extremities injection. In all our cases, there was no need for repeat CM injection.

The acquisition window was set as 40–40% of the R-R interval, and a cardiac examination was completed within 3–4 axial scans. The tube voltage was set at 80 kV, and automatic tube current regulation technology (CARE Dose4D) was applied to control the tube current, with 180 mAs as the reference. The gantry rotation time was 0.3 s, the collimation was 128 × 0.6 mm, the scan field was 250 × 250 mm, and the matrix was 512 × 512. The reconstructed layer was 0.75-mm thick with a 0.5-mm interval. Non-ionic contrast agent (350 mg I/mL, total dose 2.0 mL/kg) was injected at 0.6–4.0 mL/s, based on the child’s age, weight, and peripheral vascular conditions. Normal saline (same volume as the contrast agent) was then injected at the same rate.

### Analysis of radiation dose

After MSCT, the volume CT dose index (CTDIvol) and dose length product (DLP) were recorded, The DLP value was first multiplied by a conversion factor to fit the 16-cm phantom (the machine’s built-in DLP is based on a 32-cm phantom; the conversion factor at 80 kV was 2.3). The effective radiation dose was calculated as DLP × conversion factor × K, where K (in mSv/mGy∙cm) is the children chest specific conversion factor and was determined according to the European CT Quality Standard Guide [24] as follows: K = 0.039 for age <4 months; K = 0.026 for age 4 months to 1 year; and K = 0.018 for age 1–6 years [22].

### Evaluation of image quality

All the obtained images were evaluated independently by two radiologists with experience in cardiovascular imaging diagnosis. Any disagreements between the two radiologists in the initial evaluations were resolved by discussion. The evaluation of image quality was made using a 5-point system [25], as follows: 5 points, a clear image without any artifacts; 4 points, small artifacts present, but a high degree of diagnostic confidence remained; 3 points, blurring of the image resulting in a moderate diagnostic confidence; 2 points, part of the structure needed to be carefully distinguished, such that diagnosis was difficult; and 1 point, severe artifacts that prevented a diagnosis being made. A score of 3–5 points indicated that evaluation and diagnosis could be performed using the image, whereas a score of 1–2 points indicated that this was not possible.

### Statistical analysis

SPSS 17.0 (IBM, Armonk, NY, USA) was used for the statistical analyses. The kappa consistency test was used to measure the level of agreement between the radiologists in the assessment of image quality, with the kappa value calculated to indicate the proportion of agreement beyond that expected by chance (a kappa value of 1 indicates perfect agreement, whereas a kappa value of 0 indicates agreement no better than that expected by chance). With the surgical findings taken as the gold standard, the sensitivities, specificities, positive predictive values (PPVs), negative predictive values (NPVs), and accuracies of MSCT and TTE for the diagnostic evaluation of the major cardiac vessels were calculated. Sensitivity was calculated as the true positive rate (number of true positives divided by the sum of the number of true positives and number of false negatives); specificity as the true negative rate (number of true negatives divided by the sum of the number of true negatives and number of false positives); PPV as the number of true positives divided by the sum of the number of true positives and number of false positives; NPV as the number of true negatives divided by the sum of the number of true negatives and number of false negatives; and accuracy as the sum of the number of true positives and number of true negatives divided by the total number of results (i.e. all positives and all negatives). The chi-square test was used to compare the diagnostic accuracy between the two techniques. P < 0.05 was taken to indicate statistical significance.

## Results

### Baseline characteristics and radiation dose analysis

The baseline characteristics of these patients and the results of radiation dose analyses are presented in Table 1. CTDIvol was determined to be 0.90 ± 0.24 mGy, and DLP was determined to be 12.9 ± 4.7 mGy∙cm. The calculated ED was 0.64 ± 0.21 mSv, ranging from 0.358 mSv to 1.196 mSv.

**Table 1:** Baseline characteristics of the study participants and radiation dose analysis.

Characteristic	Value
Gender (male/female)	19/16
Age (years)	1.59 ± 1.58
Weight (kg)	9.5 ± 3.2
Heart rate (beats/min)	122±14
CTDIvol (mGy)	0.90 ± 0.24
DLP (mGy∙cm)	12.9 ± 4.7
ED (mSv)	0.64 ± 0.21

Note: CTDIvol, volume computed tomography dose index; DLP, dose length product (DLP); ED, effective radiation dose.

### Evaluation of image quality and agreement between radiologists

The mean image quality score was 4.3 ± 0.5 points. The image quality score met the requirements for diagnosis (i.e. score ≥ 3 points) in all 35 patients; there were 14 cases (40.0%) with 5 points, 20 cases (57.1%) with 4 points, and one case (2.9%) with 3 points. The kappa test for consistency determined the weighted kappa value to be 0.785 (P < 0.05), indicating substantial agreement between radiologists in the assessment of image quality.

### Comparison of MSCT and TTE for the diagnosis of CHD anomalies

Surgery, performed within one week after TTE and MSCT, confirmed the presence of 107 abnormalities and anomalous connections in the 35 patients, including 58 intracardiac abnormalities, 24 abnormal connections between the heart and major vessels, and 25 extracardiac abnormalities. Details of the abnormalities and anomalous connections detected by surgery, MSCT, and TTE in the 35 patients are shown in Table 2.

**Table 2:** Comparison of multi-slice spiral computed tomography and trans-thoracic echocardiography for the diagnosis of congenital heart malformations identified by surgery.

Malformation type	Surgery	MSCT				TTE			
		TP	TN	FP	FN	TP	TN	FP	FN
**Intracardiac malformations**									
Ventricular septal defect	23	23	12	0	0	23	12	0	0
Atrial septal defect	14	13	20	1	1	12	21	0	2
Dextrocardia	1	1	34	0	0	1	34	0	0
Single atrium	1	1	34	0	0	1	34	0	0
Single ventricle	2	2	33	0	0	2	33	0	0
Cor triatrium sinistrum	2	2	33	0	0	2	33	0	0
Endocardial cushion defect	1	1	34	0	0	1	34	0	0
Tricuspid valve atresia	1	1	34	0	0	1	34	0	0
Right ventricular outflow tract stenosis	13	13	22	0	0	13	22	0	0
**Heart-great vessel connection anomalies**									
Transposition of great arteries	2	2	33	0	0	2	33	0	0
Double outlet right ventricle	1	1	34	0	0	1	34	0	0
Aortic saddle	16	16	19	0	0	16	19	0	0
Anomalous pulmonary venous connection	2	2	33	0	0	2	32	1	0
Persistent left superior vena cava	3	3	32	0	0	3	32	0	0
**Extracardiac vascular abnormalities**									
Pulmonary artery stenosis	5	5	30	0	0	3	30	0	2
Pulmonary artery atresia	3	3	32	0	0	3	32	0	0
Bicuspid pulmonary valve malformation	1	1	34	0	0	1	34	0	0
Subvalvular aortic stenosis	1	1	34	0	0	1	34	0	0
Patent ductus arteriosus	12	10	23	0	2	9	23	0	0
Vagus left subclavian artery	1	1	34	0	0	0	34	0	0
Anomalous origin of coronary artery	2	2	33	0	0	0	33	0	0
**Total**	107	104	627	1	3	97	627	1	10

Note: FN, false negatives; FP, false positives; MSCT, multi-slice spiral computed tomography; TN, true negatives; TP, true positives; TTE, trans-thoracic echocardiography.

The total number of cardiovascular malformations detected by MSCT and TTE were 104 and 97, respectively, with the overall sensitivity of MSCT (97.2%) being significantly higher than that of TTE (90.6%; χ^2^ = 4.013, P < 0.05). The sensitivity of MSCT for the diagnosis of intracardiac malformations was 98.2% (57/58), not significantly different from the value of 96.6% (56/58) for TTE (χ^2^ = 0.342, P > 0.05). Both imaging modalities successfully identified all cases of anomalous connections between the heart and great vessels (24/24). The sensitivity of MSCT for the diagnosis of extracardiac great vessel malformations was 92.0% (23/25), substantially higher than the value of 68.0% (17/25) for TTE (χ^2^ = 4.500, P < 0.05). The overall sensitivity, specificity, PPV and NPV were 97.2%, 99.8%, 99.0%, and 99.5%, respectively, for MSCT, and 90.6%, 99.8%, 99.0%, and 98.4%, respectively, for TTE.

### Case presentation

**Fig 1:**
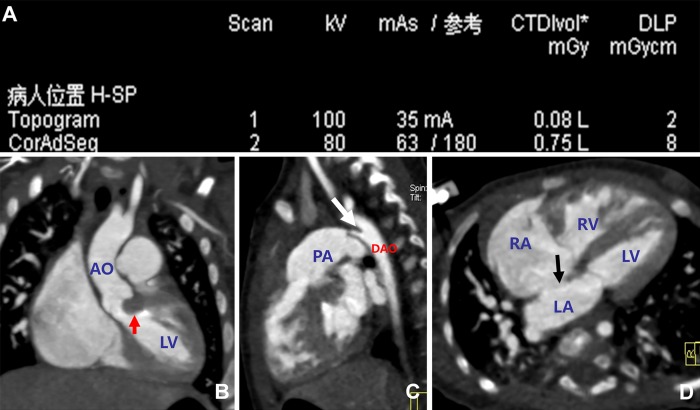
Representative multi-slice spiral computed tomography images from a female patient aged 4 months. A. Scanning parameters. CTDIvol, volume computed tomography dose index; DLP, dose length product (DLP). The effective radiation dose was calculated to be 0.026 × 2.3 × 8 = 0.478 mSv. B-D. Multiplanar reconstructed images showing subvalvular aortic stenosis, with one side of the ventricular septum protruding into the left ventricular outflow tract (B, red arrow). It was observed in the aortic isthmus that one tubular channel was connected to the aorta (C, white arrow), and there was an atrial septal defect (D, black arrow). AO, aorta; DAO, descending aorta; LA, left atrium; LV, left ventricle; PA, pulmonary artery; RA, right atrium; RV, right ventricle.

Fig 1 shows representative MSCT images from a female patient aged 4 months, for who the ED was calculated to be only 0.478 mSv. The multiplanar reconstructed (MPR) images clearly demonstrated subvalvular aortic stenosis, with one side of the ventricular septum protruding into the left ventricular outflow tract. In addition, in the aortic isthmus it was observed that one tubular channel was connected to the aorta; an atrial septal defect was also evident.

**Fig 2:**
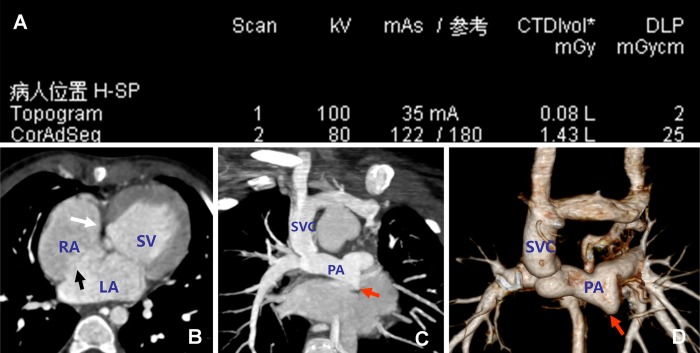
Representative multi-slice spiral computed tomography images from a male patient aged 3 years. A. Scanning parameters. CTDIvol, volume computed tomography dose index; DLP, dose length product (DLP). The effective radiation dose was calculated to be 0.018×2.3×25 = 1.035 mSv. B. Multiplanar reconstructed image showing a single ventricle, tricuspid valve atresia (white arrow), and a large atrial septal defect (black arrow). C. Maximum intensity projection showing the connection between the superior vena cava and right pulmonary artery (after surgery with the Glenn procedure), and primary pulmonary artery atresia (red arrow). D. Volume rendered image showing the features seen in C. LA, left atrium; PA, pulmonary artery; RA, right atrium; SV, single ventricle; SVC, superior vena cava.

Fig 2 presents representative images from a 3-year-old male patient, for who the ED was 1.035 mSv. A variety of anomalies were clearly detectable using MSCT, including a single ventricle, tricuspid valve atresia, and a large atrial septal defect. After performance of the Glenn procedure, a connection between the superior vena cava and right pulmonary artery could be seen, and primary pulmonary artery atresia was observed.

**Fig 3:**
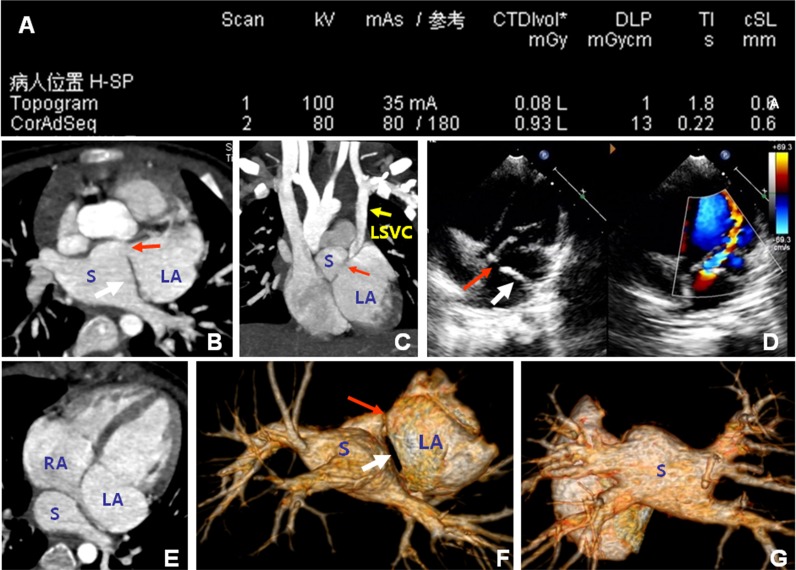
Representative multi-slice spiral computed tomography images from a female patient aged 2 years. A. Scanning parameters. CTDIvol, volume computed tomography dose index; DLP, dose length product (DLP). The effective radiation dose was calculated to be 0.018×2.3×13 = 0.538 mSv. The maximum density in B, C, and E revealed a membrane in the left atrium (white arrow), which separated the left atrium into the left atrium proper (LA) and a split atrium (S). Non-continuous perforated holes existed on the anterior part of the membrane (red arrow). The left superior vena cava is shown (yellow arrow). D. TTE revealed the membrane in the left atrium (white arrow) and communicating holes (red arrows). F and G. VR images show that the membrane separated the atrium into two chambers. The communicating holes were present at the anterior part of the membrane, and that left and right pulmonary veins were led to the split atrium (S). LA, left atrium; S, split atrium; RA, right atrium; LSVC, left superior vena cava; TTE, trans-thoracic echocardiography; VR, volume rendered.

Fig 3 presents representative images from a 2-year-old male patient, for who the ED was 0.538 mSv. The main feature in this patient was the separation of the left atrium by a septum, resulting in a 2-section atrium. Both MSCT and TTE revealed this membrane.

## Discussion

The main findings of the present study were that prospective ECG-gated MSCT was able to diagnose complex CHD with a sensitivity and specificity of 97.2% and 99.8%, respectively. Furthermore, the sensitivity of MSCT was superior to that of TTE overall, and was considerably higher than that of TTE for the diagnosis of extracardiac great vessel malformations (92.0% vs. 68.0%). In addition, accurate imaging of intracardiac malformations, anomalous connections between the heart and great vessels, and extracardiac great vessel malformations by MSCT was achieved for a mean radiation exposure of only 0.64 mSv. In view of the advantages of MSCT over MRA, including superior spatial resolution, much shorter scanning time and ability to visualize the lung parenchyma and airways, we suggest that MSCT should be considered as a useful complementary imaging technique to TTE, particularly in patients with suspected complex CHD.

CHD is a common cardiac disease in infants and young children, affecting approximately 1% of all live births [1], with complex CHD accounting for around 50% of the cases. Accurate and comprehensive preoperative evaluation of complex CHD is critical for the selection of the appropriate surgical approach and prognostic evaluation. MSCT has the advantages of fast scanning speed, high resolution, powerful image post-processing, and clear visualization of the anatomic structures of the cardiac chambers and great vessels [3, 4]. The sensitivity of children to X-rays is higher than that of adults, such that their risk of complications associated with radiation exposure is also greater than that of adults [13, 26]. Although decreasing the tube voltage and current can effectively reduce the dose of radiation received by a patient, the level of radiation exposure in children can remain high in the absence of prospective ECG-gating.

Prospective ECG-gated imaging, in which scanning takes place only at a specific part of the cardiac cycle, can reduce the radiation dose as compared with ungated or retrospective ECG-gated scanning [17, 27–29], and this technique has been widely applied in CTA [16–23, 27–29]. In the present study, the acquisition window was set as 40–40% of the R-R interval to further decrease the radiation dose. For the 35 children in this study, the average ED was only 0.64 ± 0.21 mSv, comparable to values reported previously for prospective ECG-gated MSCT of children with CHD [16–18, 20–22, 27, 29]. The malformations in complex CHD can vary substantially among children, and we have observed differences in contrast agent filling time among patients. An empirical delay method was adopted in this study, and the delay time (21–23 s) was set based on the different contrast agent injection sites. After circulating for a certain period of time, uniform development of the contrast agent was observed in the various cardiac chambers and great vessels, and the structures could be clearly delineated. Inadequate contrast agent filling or hardened artifacts caused by inappropriate triggering time in the conventional bolus chasing method were avoided. Our technique had good repeatability, and re-scanning was not required in any patients. The images from all 35 children in this study met the diagnostic requirements, and the average image score was 4.3 ± 0.5.

An important finding of this study was that although the diagnostic capabilities of MSCT and TTE were similar for the detection of intracardiac malformations or anomalous heart-great vessel connections, MSCT was far superior to TTE in the diagnosis of extracardiac great vessel malformations (sensitivity of 92.0% for MSCT, 68.0% for TTE). This latter finding is consistent with those of previous investigations; for example, Nie et al. reported a sensitivity of 97.5% for dual-source CT and 79.6% for TTE in the diagnosis of extracardiac vascular anomalies [30], while in a similar study, Xu et al. obtained values of 98.8% and 62.5%, respectively[31]. On the other hand, when comparing DSCT vs. TTE, Cheng et al. [22] showed that both approaches had similar sensitivity and specificity. Thus, the use of TTE alone could lead to an under-diagnosis of extracardiac great vessel malformations, as evidenced by the numerous cases of missed diagnosis found in this study. This emphasizes the potential role for MSCT in the accurate diagnosis of complex CHD.

Using MSCT, missed diagnoses occurred for only three of the 107 abnormalities (2.8%), compared with 10 (9.3%) for TTE. The missed diagnoses for MSCT were one case of small atrial septal defect and two cases of patent ductus arteriosus. Notable limitations of MSCT were that it only displayed static images, poorly visualized valvular structures, and was unable to display hemodynamic changes. In contrast, TTE was highly sensitive at detecting blood shunting and reflux, and could dynamically display, in real-time, various valves and other intracardiac structures. In view of this, we suggest that MSCT and TTE should be used as complementary techniques for the evaluation of intracardiac structures. The combination of both approaches would also allow evaluation of the lungs in children with CHD (a strength of MSCT but not TTE), thereby providing comprehensive and accurate information to facilitate the planning of surgery.

The present study has certain limitations. First, only a small number of patients were included in the analysis. Second, direct comparisons of diagnostic capability were not made with other imaging techniques, such as MRA. Third, the radiation dose was not directly measured but was estimated on the basis of the scanning parameters and assumptions regarding conversion factors; however, this is an approach commonly used in this type of study. Fourth, MSCT was unable to provide hemodynamic and functional information data that would facilitate the evaluation of extracardiac vascular abnormalities. Further larger-scale, prospective studies with additional comparator groups are needed to confirm and extend our findings.

In conclusion, prospective ECG-gated MSCT was able to accurately visualize intracardiac malformations, anomalous connections between the heart and great vessels, and extracardiac great vessel malformations, for a mean radiation exposure of only 0.64 mSv. Furthermore, MSCT was superior to TTE in the diagnosis of extracardiac vascular malformations. We suggest that MSCT could be used as a complementary technique to TTE to improve the characterization of complex CHD before surgery.

## Supporting Information

S1 Data Raw data. (XLS)
